# Spatial Patterns and Determinants of Childhood Anemia in the Democratic Republic of Congo: Evidence From the 2023/2024 DHS

**DOI:** 10.1155/anem/6657161

**Published:** 2026-09-30

**Authors:** Zelalem Meraf Wolde, Nigussie Adam Birhan, Kefale Tilahun Getahun, Kahsay Kiros Hailu, Batholomew Chibuike James, Roy Rillera Marzo, Wegayehu Enbeyle Sheferaw, Endale Alemayehu Ali, Denekew Bitew Belay

**Affiliations:** ^1^ Department of Statistics College of Natural and Computational Science Injibara University Injibara Ethiopia inu.edu.et; ^2^ Department of Statistics College of Natural and Computational Science University of Gondar Gondar Ethiopia uog.edu.et; ^3^ Public Health Program School of Professional Studies Angeles University Foundation Angeles Pampanga Philippines; ^4^ Faculty of Humanities and Health Sciences Curtin University Malaysia Miri Sarawak Malaysia; ^5^ Faculty of Public Health Universitas Airlangga Surabaya East Java Indonesia unair.ac.id; ^6^ Health Research Institute University of Canberra Australian Capital Territory (ACT) Bruce Australia canberra.edu.au; ^7^ Department of Statistics College of Natural and Computational Science Mizan-Tepi University Tepi Ethiopia mtu.edu.et; ^8^ Department of Public Health and Primary Care KU Leuven Kapucijnenvoer 33 3000 Leuven Belgium kuleuven.be; ^9^ Department of Statistics College of Science Bahir Dar University Bahir Dar Ethiopia bdu.edu.et; ^10^ School of Health Systems and Public Health Faculty of Health Sciences University of Pretoria Pretoria South Africa up.ac.za

**Keywords:** childhood anemia, Democratic Republic of Congo, determinants, multilevel analysis, spatial distribution

## Abstract

**Background:**

Anemia in children is a serious public health issue in low‐income settings, particularly sub‐Saharan Africa. In the Democratic Republic of Congo (DRC), evidence on the geographic distribution and multilevel determinants of childhood anemia remains limited. This study aimed to examine the spatial pattern and identify individual, household, and community‐level determinants of anemia among children aged 6–59 months in the DRC.

**Methods:**

A secondary analysis was conducted using data from the 2023/2024 DRC‐DHS. A weighted sample of 9449 children aged 6–59 months was included. Spatial analyses were performed using Global Moran’s I, Getis‐Ord Gi∗, and Kriging interpolation techniques to assess geographic clustering and spatial patterns of childhood anemia. Multilevel binary logistic regression models were fitted to identify factors associated with childhood anemia, and adjusted odds ratios (AORs) with 95% confidence intervals (CIs) were reported.

**Results:**

Of the 9449 children, 6092 (64.5%) were anemic. Spatial analysis showed significant geographic clustering of anemia in the DRC (Moran’s I = 0.516, *p* = 0.0078), with hotspot regions in western‐central and cold spots in eastern provinces. Our findings showed that several factors were associated with lower odds of childhood anemia. Specifically, we observed reduced odds among children aged 24–35 months (AOR = 0.48; 95% CI: 0.34–0.68), females (AOR = 0.89; 95% CI: 0.81–0.98), and those from households in the middle wealth index (AOR = 0.86; 95% CI: 0.75–0.98). In contrast, higher odds of childhood anemia were observed among children with severe stunting (AOR = 1.19; 95% CI: 1.04–1.37), severe underweight (AOR = 1.58; 95% CI: 1.31–1.92), fever (AOR = 1.24; 95% CI: 1.10–1.40), those born to anemic mothers (AOR = 1.39; 95% CI: 1.26–1.53), children from households with female household head (AOR = 1.21; 95% CI: 1.08–1.35), and those residing in communities with higher levels of poverty (AOR = 1.24; 95% CI: 1.05–1.47).

**Conclusion:**

Childhood anemia in the DRC remains highly prevalent and spatially clustered, with western and northern provinces particularly affected. Individual, household, and community factors were identified as significant determinants of anemia risk. Targeted interventions focusing on high‐risk regions, nutritional support, maternal health, and socioeconomic improvements are critical to reducing childhood anemia in the DRC.

## 1. Introduction

Anemia continues to be a serious public health concern worldwide, disproportionately impacting populations in low‐ and middle‐income nations [1, 2]. Anemia is defined as a state in which the concentration of hemoglobin (Hb) is less than what is physiologically required to enable efficient oxygen transport to bodily tissues [3]. Childhood anemia is defined by the WHO as Hb < 11 g/dL in children aged 6–59 months [4–6]. The primary cause of anemia is iron deficiency, and the other significant factors include Hb abnormalities, infections, chronic inflammation, vitamin deficiencies, and insufficient food intake [7].

Globally, anemia affects approximately 273 million children aged 6–59 months, representing about 40% of the world’s preschool population [8, 9]. Childhood anemia remains highly prevalent in Africa and sub‐Saharan Africa (SSA), posing a major public health challenge. According to a 2022 comprehensive research, the region’s pooled prevalence among children ages 6 to 59 months was 59% [10]. In SSA, approximately 64.1% of children aged 6–59 months are affected [11]. Widespread malaria, parasitic infections, long‐term food instability, and restricted access to high‐quality healthcare services are the main causes of this high burden, underscoring the critical need for focused measures to reduce anemia and its related health effects [12]. Early childhood anemia causes problems for both individuals and society as a whole. It is linked to delayed motor skills, poor academic performance, increased susceptibility to infections, impaired physical and cognitive development, and higher morbidity and mortality [13–15].

For children between the ages of 6 and 59 months, childhood anemia continues to be a serious public health concern in the Democratic Republic of Congo (DRC). According to a survey conducted in the DRC, children between the ages of 6 and 59 months had a significant frequency of anemia, 68.1% in rural regions [16] and almost 65% in Kinshasa, the country’s capital [17]. Childhood anemia prevalence in the region consistently exceeds the WHO threshold of 40%, classifying it as a severe public health problem [18]. Anemia patterns are significantly influenced by a number of factors, including poverty, the environment, living conditions, and child feeding practices, as seen by the DRC’s persistent regional and socioeconomic discrepancies [19].

Various factors contributing to childhood anemia have been extensively documented in previous studies, including household and maternal factors (low wealth, large family size, low maternal education, unemployment, inadequate dietary diversity, maternal anemia), child‐related factors (young age, stunting, wasting, recent fever, diarrhea, malaria, low birth weight) [20], environmental and community factors (poor sanitation, unsafe water, indoor air pollution, geographic location), and parasitic infections (soil‐transmitted helminths, schistosomiasis), with malaria being a major driver in endemic areas of Central and East Africa [20–23].

The DRC has implemented a number of maternal and child health treatments, such as food fortification programs, vitamin supplementation, deworming programs, and malaria control measures, yet the rate of childhood anemia is still unacceptably high [24]. Childhood anemia has been associated to significant individual and household‐level determinants in the DRC [16]. However, limited evidence exists on its spatial patterns, geographic clustering, and the combined influence of individual‐, household‐, and community‐level determinants.

Understanding the spatial distribution of childhood anemia is particularly important in the DRC because the country has substantial geographic, socioeconomic, and healthcare access inequalities [25]. Spatial clustering of anemia may reflect shared environmental exposures, differences in healthcare availability, nutritional conditions, and community‐level vulnerabilities. Therefore, integrating spatial analysis with multilevel modeling can provide a more comprehensive understanding of where anemia hotspots exist and which factors contribute to childhood anemia at different levels. Spatial statistical techniques such as Moran’s I, Getis‐Ord Gi∗, and spatial interpolation methods can identify geographic patterns and high‐risk areas, supporting targeted and context‐specific interventions [26, 27]. Therefore, this study aims to assess the spatial distribution and multilevel determinants of anemia among children aged 6–59 months in the DRC using nationally representative DHS data. By integrating spatial analysis with multilevel modelling, this study provides evidence to identify high‐risk areas and inform geographically targeted strategies for childhood anemia prevention and control in the DRC.

## 2. Methods

### 2.1. Study Design and Study Setting

A cross‐sectional study was conducted using data from the 2023/2024 Democratic Republic of Congo Demographic and Health Survey (DRC‐DHS). DRC is the second largest nation in Africa, after Algeria. The DRC covers the majority of the Congo River basin and extends from the Atlantic Ocean to the eastern plateau. Located in Central Africa, it shares borders with nine other countries, including the enclave of Cabinda, and is traversed by the equator [28]. The DRC covers an area of approximately 2,344,858 km [29]. The DRC is administratively divided into 26 provinces [30] with a projected population of over 104 million in 2023, approximately 17% of whom are children aged 6–59 months.

### 2.2. Sample Size and Sampling Procedures

This study utilized secondary data from the DRC‐DHS 2023/24, which comprised a weighted sample of 9449 children aged 6–59 months. The DRC‐DHS covered both urban and rural regions in each of the 26 provinces using a stratified two‐stage cluster methodology. The first stage involved selecting enumeration areas using probability proportional to size (PPS) sampling. In the second stage, a systematic sampling technique was used to select households within each selected EA. The DHS sampling design included stratification by residence and administrative regions to ensure nationally representative estimates. All children in the DRC between the ages of 6 and 59 months served as the study population.

#### 2.2.1. Inclusion Criteria

Children between the ages of 6 and 59 months who had complete data on the variables used in the analysis from the chosen enumeration areas and Hb measurements were included in the study.

#### 2.2.2. Exclusion Criteria

Children aged 6–59 months who did not have Hb measurements were not assessed for anemia according to DHS procedures, or had missing information on the outcome variable were excluded. Missing values for predictor variables were handled using complete‐case analysis.

### 2.3. Study Population

The study population comprised children aged 6–59 months living in the selected enumeration areas, which served as the primary sampling units. Mothers or caregivers were interviewed, and for those with more than one eligible child, information was collected on the most recent child.

## 3. Variables of the Study

### 3.1. Outcome Variable

The primary outcome was anemia status among children aged 6–59 months. Hb concentration was measured from finger‐ or heel‐prick blood samples using a portable HemoCue analyzer, and values were adjusted for altitude following DHS procedures. According to WHO standards, children with Hb levels less than 11 g/dL were considered anemic, whereas those with levels greater than 11 g/dL were considered nonanemic [31].

### 3.2. Predictor Variables

Child, mother, household, and community‐level characteristics were among the potential predictors that were chosen based on previous studies and DHS data [32–35]. Child‐level factors were age, sex, birth size, stunting, wasting, underweight, birth order, and recent illnesses (diarrhea, fever, and cough). Maternal and household factors included maternal age, education, working status, marital status, media exposure, husband’s education, household size, number of under‐five children, wealth index, and access to water, sanitation, and cooking fuel. Community‐level factors comprised place of residence, region, cluster altitude, and aggregated measures of community women’s education, poverty, and media exposure. Community‐level variables were created by aggregating individual and household characteristics within each enumeration area. Community poverty was derived from the household wealth index (poor, middle, and rich), community education from women’s educational attainment (no education, primary, and secondary or higher), and community media exposure from women’s exposure to at least one mass media source (newspaper, radio, or television). The national median value served as the cut‐off point for classifying the aggregated variables as low or high.

### 3.3. Data Management and Analysis

Data were processed and analyzed using Stata 14, while ArcGIS 10.8 was employed for spatial analyses. The DHS sampling weight (V005), primary sampling units, and stratification variables were applied to account for the complex survey design and ensure representative and reliable estimates [36]. All analyses incorporated DHS sampling weights and accounted for clustering and stratification to produce unbiased population‐level estimates.

### 3.4. Spatial Analysis

Geographical patterns and clustering of anemia were investigated using spatial analyzes in ArcGIS 10.8, which also revealed regions of high prevalence among children in the DRC aged 6 to 59 months [37]. In order to determine high‐burden areas in requiring targeted interventions and to evaluate the geographic clustering of anemia cases, spatial analysis was carried out.

To evaluate the general spatial pattern of childhood anemia and determine whether its distribution was random, scattered, or clustered, Global Moran’s I was employed [38]. Moran’s I has a range of −1 to +1, where values close to zero indicate a random spatial pattern, negative values indicate dispersion, and positive values indicate clustering [39]. A statistically significant Moran’s I (*p* < 0.05) confirms the presence of nonrandom spatial clustering of anemia cases, although it does not specify the exact cluster locations [40, 41].

Getis–Ord Gi∗ statistics were used to identify locations with significant spatial clusters. This analysis identified significant anemia hotspots and cold spots. Enumeration areas with statistically significant positive Gi values were classified as hotspots, while areas with significant negative Gi values were classified as cold spots based on their corresponding Z‐scores and *p* values [42, 43].

Ordinary Kriging interpolation was used to estimate anemia prevalence in unsampled areas. This method was selected because it incorporates spatial autocorrelation between sampled locations to predict anemia prevalence in areas without direct observations and generate a continuous spatial surface of anemia distribution across the country [44].

### 3.5. Multilevel Logistic Regression Analysis

The hierarchical structure of the DHS data, where children are nested within clusters and produce associated observations, was taken into consideration using multilevel modeling. A random intercept two‐level mixed‐effects logistic regression model was constructed, with children serving as level‐one units and enumeration regions serving as level‐two units. This approach accounts for the correlation of observations within clusters and allows simultaneous assessment of individual‐, household‐, and community‐level determinants while controlling for unexplained cluster‐level heterogeneity [45].

To account for potential confounding, variables with *p* < 0.25 in the bivariable analysis were added to the multivariable model. Adjusted odds ratios (AORs) with 95% confidence intervals (CIs) were reported, with *p* < 0.05 indicating statistical significance [46].

### 3.6. Model Building and Selection

A stepwise model‐building approach was applied to assess the contribution of different levels of determinants and quantify unexplained cluster‐level variation. Model I (null model) included only the random intercept and was used to estimate baseline variability among clusters [47]. Individual‐level factors were evaluated by Model II, community‐level factors were investigated by Model III, and variables from both levels were merged in the final model by Model IV. The intraclass correlation coefficient [48] was calculated to determine the proportion of total variance in childhood anemia attributable to differences between clusters [48]. Cluster‐level variation was assessed using the median odds ratio (MOR), while the proportional change in variance (PCV) was used to determine the change in unexplained variance following the inclusion of covariates.

(1) ICC=σμ02σμ02+π2/3,MOR=exp  2×σμ02×0.6745,PCV=VA−VBVA×100,

where variance *π* ^2^/3 = 3.29 and $\sigma_{\mu 0}^{2}$ is the variance of the higher level (region) [49].

Model performance was compared using the likelihood ratio test, AIC, and BIC, with the best‐fitting model selected [50].

## 4. Result

The study included 9449 children aged 6–59 months, of whom 6092 (64.5%) were anemic, while 3357 (35.5%) were not anemic. This means that more than half of children aged 6–59 months were anemic in Congo, as shown in Figure 1.

**FIGURE 1:**
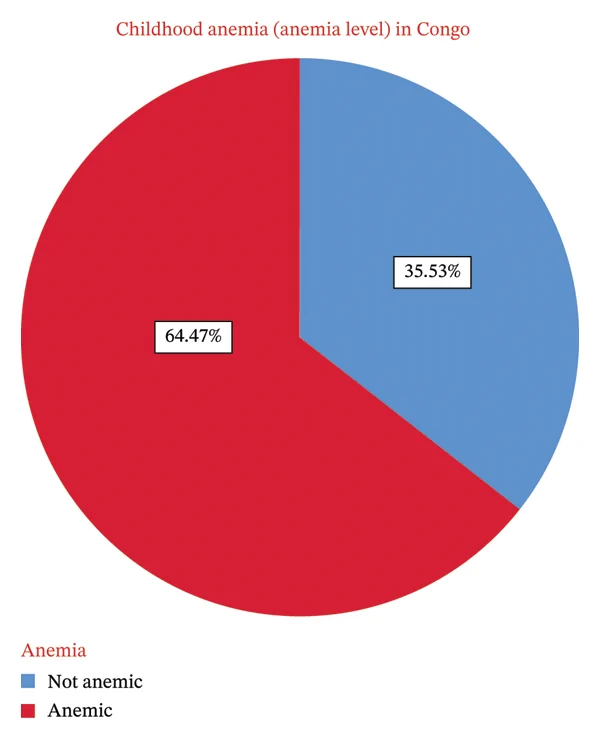
Childhood anemia in the Democratic Republic of Congo.

Among the 9449 sampled children aged 6–59 months, the majority, 4824 (51.05%), were male, while 4625 (48.95%) were female. Regarding childhood stunting, 4780 (50.58%) participants were classified as not stunted, 2234 (23.64%) as moderately stunted and 2435 (25.77%) as severely stunted. Regarding recent health conditions, 7278 (77.0%) children had not experienced fever in the 2 weeks preceding the survey, whereas 2171 (23.0%) had experienced fever during the same period, as shown in Table 1.

**TABLE 1:** Frequency and total percentage of child characteristics in relation to childhood anemia (DRC‐DHS 2023/24).

Variables	Categories	Anemia status		Frequency (%)	*X* ^2^ (*p* value)
		Not anemic	Anemic		
Child age (months)	6–11	256 (2.7)	844 (8.93)	1100 (11.64)	< 0.001
	12–23	606 (6.41)	1502 (15.89)	2108 (22.30)	
	24–35	761 (8.06)	1326 (14.03)	2087 (22.08)	
	36–47	847 (8.96)	1262 (13.36)	2109 (22.31)	
	48–59	887 (9.38)	1158 (12.25)	2045 (21.63)	

Sex of child	Male	1646 (17.42)	3178 (33.63)	4824 (51.05)	0.004
	Female	1711 (18.10)	2914 (30.84)	4625 (48.95)	

Stunting	Not stunted	1799 (19.03)	2981 (31.55)	4780 (50.58)	< 0.001
	Moderately stunted	802 (8.49)	1432 (15.15)	2234 (23.64)	
	Severely stunted	756 (8.00)	1679 (17.76)	2435 (25.77)	

Underweight	Not underweight	2562 (27.10)	4182 (44.24)	6744 (71.34)	< 0.001
	Moderate underweight	564 (5.97)	1251 (13.23)	1815 (19.20)	
	Severe underweight	231 (2.44)	659 (6.97)	890 (9.41)	

Wasting	Not wasted	3159 (33.44)	5573 (59.03)	8732 (92.47)	< 0.001
	Moderate wasting	136 (1.44)	372 (3.93)	508 (5.38)	
	Severe wasting	62 (0.66)	147 (1.55)	209 (2.21)	

Diarrhea	No	2928 (30.98)	5074 (53.69)	8002 (84.67)	< 0.001
	Yes	429 (4.54)	1018 (10.77)	1447 (15.31)	

fever,	No	2710 (28.66)	4568 (48.32)	7278 (76.98)	< 0.001
	Yes	647 (6.84)	1524 (16.13)	2171 (22.97)	

cough	No	2628 (27.80)	4777 (50.55)	7405 (78.35)	0.883
	Yes	729 (7.71)	1315 (13.91)	2044 (21.63)	

Vitamin A supplementation	No	1696 (17.95)	3243 (34.32)	4939 (52.27)	0.012
	Yes	1661 (17.58)	2849 (30.14)	4510 (47.73)	

Type of birth	Single	3264 (34.54)	5919 (62.60)	9183 (97.14)	0.845
	Multiple	93 (0.98)	173 (1.83)	266 (2.82)	

Total children ever born	1–2	833 (8.81)	1538 (16.28)	2371 (25.09)	0.521
	3–4	1043 (11.04)	1940 (20.52)	2983 (31.56)	
	5+	1481 (15.67)	2614 (27.67)	4095 (43.34)	

Among the 9449 mother–child pairs included in the study, maternal educational status varied considerably, with 4117 (43.6%) of mothers having attained secondary or higher education, 3226 (34.1%) having primary education, and the rest 2106 (22.3%) having no formal education. In terms of maternal anemia status, 3922 (41.5%) of mothers were anemic, while 5527 (58.5%) were not, indicating a substantial proportion of mothers at risk of nutritional deficiencies that could affect child health.

Regarding household wealth, more than half of the participants, 5250 (55.6%), belonged to poor, followed by 2143 (22.7%) in middle‐income and the rest 2056 (21.8%) in rich households, as shown in Table 2.

**TABLE 2:** Frequency and total percentage of maternal and household characteristics for childhood anemia (DRC‐DHS 2023/24, *N* = 9449).

Variables	Categories	Anemia status		Frequency (%)	*X* ^2^ (*p* value)
		Not anemic	Anemic		
Mother age	15–19	106 (1.12)	280 (2.96)	386 (4.09)	0.004
	20–24	769 (8.14)	1446 (15.29)	2215 (23.44)	
	25–29	751 (7.95)	1377 (14.57)	2128 (22.52)	
	30–34	700 (7.40)	1224 (12.95)	1924 (20.36)	
	35–39	625 (6.61)	1052 (11.13)	1677 (17.75)	
	40–44	296 (3.13)	559 (5.91)	855 (9.05)	
	45–49	110 (1.16)	154 (1.63)	264 (2.79)	

Mother’s education	No education	683 (7.23)	1423 (15.06)	2106 (22.29)	< 0.001
	Primary	1085 (11.48)	2141 (22.66)	3226 (34.14)	
	Secondary and higher	1589 (16.81)	2528 (26.74)	4117 (43.57)	

BMI category	Underweight	431 (4.56)	989 (10.46)	1420 (15.03)	< 0.001
	Normal	2349 (24.86)	4334 (45.87)	6683 (70.73)	
	Overweight	443 (4.69)	595 (6.30)	1038 (10.99)	
	Obese	134 (1.42)	174 (1.84)	308 (3.26)	

Mother occupation	Not working	1222 (12.93)	2148 (22.73)	3370 (35.67)	0.267
	Working	2135 (22.59)	3944 (41.72)	6079 (64.33)	

Marital status of mother	Single	116 (1.23)	225 (2.38)	341 (3.61)	0.035
	Married	2993 (31.67)	5331 (56.42)	8324 (88.09)	
	Widowed	41 (0.43)	115 (1.22)	156 (1.65)	
	Divorced	207 (2.19)	421 (4.45)	628 (6.65)	

Mother anemia status	Not anemic	2157 (22.82)	3370 (35.66)	5527 (58.49)	< 0.001
	Anemic	1200 (12.70)	2722 (28.80)	3922 (41.51)	

Wealth index	Poor	1630 (31.05)	3620 (68.95)	5250 (55.56)	< 0.001
	Middle	797 (37.19)	1346 (62.81)	2143 (22.68)	
	Rich	930 (45.23)	1126 (54.77)	2056 (21.76)	

Toilet facility	Unimproved/none	1268 (36.51)	2205 (63.49)	3473 (36.76)	0.128
	Improved	2089 (34.96)	3887 (65.04)	5976 (63.24)	

Religion	Christian	794 (36.59)	1376 (63.41)	2170 (22.97)	0.206
	Muslim	1087 (36.10)	1924 (63.90)	3011 (31.87)	
	Other	1476 (34.58)	2792 (65.42)	4268 (45.17)	

Household head sex	Male	2574 (36.45)	4488 (63.55)	7062 (74.74)	0.001
	Female	783 (32.80)	1604 (67.20)	2387 (25.26)	

Distance to health facility	Not a big problem	1958 (36.56)	3397 (63.44)	5355 (56.67)	0.016
	Big problem	1399 (34.17)	2695 (65.83)	4094 (43.33)	

Partner’s education	No education	294 (34.19)	566 (65.81)	860 (9.10)	0.003
	Primary	491 (32.01)	1043 (67.99)	1534 (16.23)	
	Secondary/higher	2572 (36.46)	4483 (63.54)	7055 (74.66)	

Anemia was more common among rural children (4721; 50.0%) than in urban children (1371; 14.5%), with the majority of the 9449 children in the research living in rural areas (7108; 75.2%) compared to 2341 (24.8%) in urban settings. In terms of community poverty, 4769 (50.5%) of the children lived in low‐poverty communities, while 4680 (49.5%) were from high‐poverty areas. Anemia was more common in high‐poverty communities (34.3%) than in low‐poverty communities (30.2%), as shown in Table 3.

**TABLE 3:** Community‐level characteristics on childhood anemia in the DRC.

Variables	Categories	Anemia status		Frequency (%)	*X* ^2^ (*p* value)
		Not anemic	Anemic		
Residence	Urban	970 (10.27)	1371 (14.52)	2341 (24.79)	< 0.001
	Rural	2387 (25.27)	4721 (50.00)	7108 (75.21)	

Community poverty	Low	1918 (20.29)	2851 (30.18)	4769 (50.46)	< 0.001
	High	1439 (15.23)	3241 (34.32)	4680 (49.54)	

Community literacy	Low	1768 (18.72)	3564 (37.72)	5332 (56.43)	< 0.001
	High	1589 (16.82)	2528 (26.76)	4117 (43.57)	

### 4.1. Spatial Analysis Results

#### 4.1.1. Spatial Autocorrelation

According to the spatial autocorrelation analysis, DRC childhood anemia are geographically clustered, with some regions showing high prevalence and others showing low prevalence. A Moran’s I of 0.516, a positive z‐score of 2.66, and a highly significant *p* value of 0.0078 all supported this clustering, indicating significant spatial variability in childhood anemia in the DRC, as shown in Figure 2.

**FIGURE 2:**
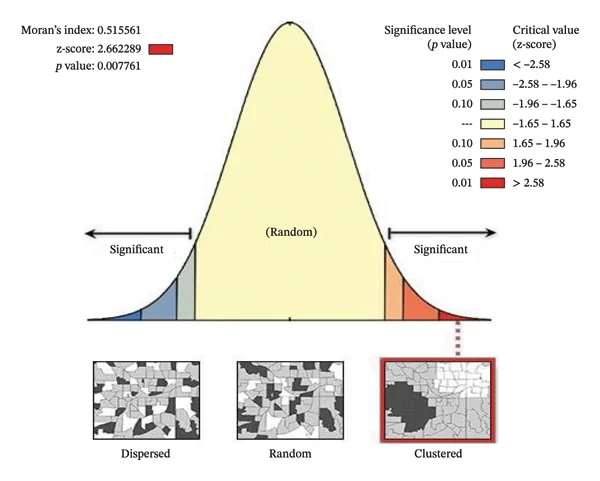
A spatial autocorrelation analysis (Moran’s I) of childhood anemia in the DRC.

There is a distinct geographic pattern in the prevalence of childhood anemia across the DRC. Hotspots, or statistically significant clusters of high anemia prevalence, were found in the western‐central provinces, especially Equateur and Tshuapa. On the other hand, Figure 3 shows the eastern regions of Nord‐Kivu and Sud‐Kivu as cold spots with significantly lower rates of childhood anemia.

**FIGURE 3:**
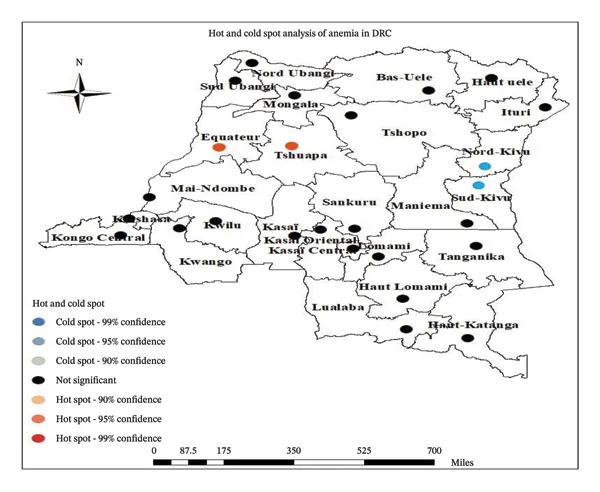
Hotspot analysis of childhood anemia in the DRC.

Significant regional differences in childhood anemia were seen throughout the DRC, according to the Kriging analysis (Figure 4). Ituri, Nord‐Kivu, and Sud‐Kivu had the lowest estimates (0.472–0.573), whereas Bas‐Uele, Équateur, Tshuapa, Mai‐Ndombe, and Kwango had the highest expected prevalence (0.717–0.818). Parts of Haut‐Lomami and Lualaba displayed intermediate expected values (0.629–0.717).

**FIGURE 4:**
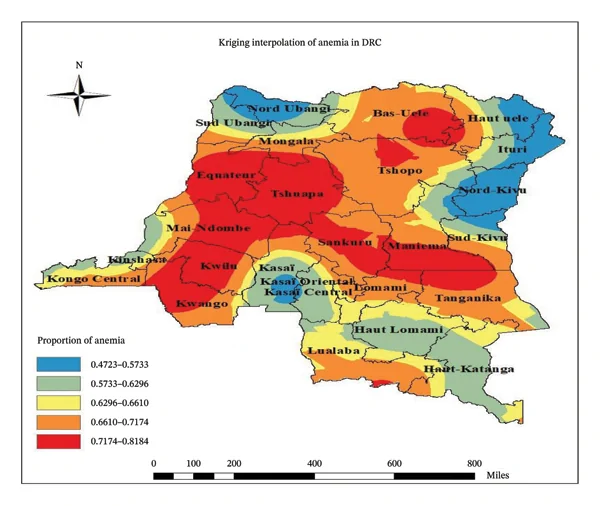
Kriging interpolation of childhood anemia in the DRC.

### 4.2. Determinants of Childhood Anemia: Multilevel Logistic Regression Analysis

Regional‐level variance decreased from 0.57 in the null model to 0.46 in Model IV, suggesting that between‐region variations in childhood anemia were significantly explained by the combined individual‐ and community‐level covariates. The interclass correlation coefficient dropped from 14.76% in the null model to 12.72% in the final model after accounting for explanatory variables, suggesting that most of the regional variation was explained. These models identified a substantial percentage of regional differences, as indicated by the PCV, which increased from 14.8% in the individual‐level model to 15.83% in the full model.

Model IV provided the best fit, reflected by an improved log‐likelihood (−5749.60) and the lowest AIC (11,567.20) and BIC (11,810.43). Model fit statistics showed a gradual improvement over the models. These results revealed that Model IV offers the most parsimonious and best‐fit explanation for the differences in childhood anemia in the DRC, as indicated in Table 4.

**TABLE 4:** Random effect analysis result and model fit statistics for childhood anemia in the DRC.

Parameters	Null model	Model II (individual‐level)	Model III (community‐level)	Model IV (full model)
Region‐level variance (SE)	0.57 (0.06)	0.49 (0.05)	0.51 (0.05)	0.48 (0.05)
ICC (%)	14.76	12.86	13.40	12.72
PCV (%)	Reference	14.80	10.63	15.83
MOR	1.66	1.60	1.62	1.60
Model fit statistics				
Log‐likelihood	−5966.58	−5751.32	−5941.02	−5749.60
AIC	11,937.17	11,572.64	11,890.04	11,567.20
BIC	11,951.47	11,823.02	11,918.65	11,810.43

#### 4.2.1. Results of Bivariate Binary Logistic Regression Analysis

Child age (months), sex of child, stunting status, underweight status, diarrhea (last 2 weeks), Vitamin A supplementation, fever (last 2 weeks), maternal education, maternal age (years), maternal BMI, maternal anemia, wealth index, toilet facility, place of residence, sex of household head, distance to health facility, and community poverty based on a *p* value of less than 0.25 were incorporated into the multivariable analysis. However, wasting, cough, type of birth, total number of child, mother occupation, marital status, religion, and community education were not included in the multivariable analysis since their *p* value was greater than 0.25.

A number of predictors were significantly related to childhood anemia in the adjusted analysis, including child age, sex, stunting status, underweight, fever, maternal education, maternal anemia, wealth index, sex of household head, and community poverty (Table 5).

**TABLE 5:** Bivariable and multivariable multilevel logistic regression analysis childhood anemia and associated risk factors in the DRC.

Variables	Categories	COR (95% CI)	*p* value	AOR (95% CI)	*p* value
Child age (months)	6–11 (Ref.)	1.00		1.00	^∗∗^
	12–23	0.77 (0.56–1.07)	0.116	0.73 (0.52–1.03)	0.072
	24–35	0.54 (0.40–0.75)	< 0.001	0.48 (0.34–0.68)	< 0.001
	36–47	0.47 (0.34–0.64)	< 0.001	0.41 (0.29–0.58)	< 0.001
	48–59	0.41 (0.30–0.56)	< 0.001	0.36 (0.25–0.50)	< 0.001

Sex of child	Male (Ref.)	1.00		1.00	^∗∗^
	Female	0.88 (0.81–0.96)	0.004	0.89 (0.81–0.98)	0.013

Stunting status	Not stunted (Ref.)	1.00		1.00	^∗∗^
	Moderately stunted	1.08 (0.97–1.20)	0.161	1.04 (0.93–1.18)	0.474
	Severely stunted	1.34 (1.21–1.49)	< 0.001	1.19 (1.04–1.37)	0.012

Underweight status	Normal (Ref.)	1.00		1.00	^∗∗^
	Moderate underweight	1.36 (1.22–1.52)	< 0.001	1.22 (1.07–1.40)	0.004
	Severe underweight	1.75 (1.49–2.05)	< 0.001	1.58 (1.31–1.92)	< 0.001

Diarrhea (last 2 weeks)	No (Ref.)	1.00		1.00	
	Yes	1.37 (1.21–1.55)	< 0.001	1.11 (0.96–1.28)	0.153

Vitamin A supplementation	No (Ref.)	1.00		1.00	
	Yes	0.90 (0.82–0.98)	0.012	0.97 (0.88–0.07)	0.548

Fever (last 2 weeks)	No (Ref.)	1.00		1.00	^∗∗^
	Yes	1.40 (1.26–1.55)	< 0.001	1.24 (1.10–1.40)	< 0.001

Maternal education	No education (Ref.)	1.00		1.00	^∗∗^
	Primary	0.95 (0.84–1.06)	0.362	0.96 (0.84–1.10)	0.532
	Secondary & above	0.76 (0.68–0.85)	< 0.001	0.85 (0.74–0.98)	0.029

Maternal age (years)	15–19 (Ref.)	1.00		1.00	
	20–24	0.71 (0.56–0.90)	0.006	0.84 (0.64–1.09)	0.185
	25–29	0.69 (0.55–0.88)	0.003	0.92 (0.70–1.20)	0.526
	30–34	0.66 (0.52–0.84)	0.001	0.85 (0.65–1.11)	0.242
	35–39	0.64 (0.50–0.81)	< 0.001	0.80 (0.61–1.05)	0.104
	40–44	0.71 (0.55–0.93)	0.013	0.89 (0.66–1.19)	0.421
	45–49	0.53 (0.38–0.74)	< 0.001	0.70 (0.48–1.01)	0.056

Maternal BMI	Underweight (Ref.)	1.00		1.00	
	Normal	0.80 (0.71–0.91)	0.001	0.95 (0.82–1.09)	0.469
	Overweight	0.59 (0.50–0.69)	< 0.001	0.91 (0.74–1.11)	0.329
	Obese	0.57 (0.44–0.73)	< 0.001	1.09 (0.81–1.46)	0.585

Maternal anemia	Not anemic (Ref.)	1.00		1.00	^∗∗^
	Anemic	1.45 (1.33–1.58)	< 0.001	1.39 (1.26–1.53)	< 0.001

Wealth index	Poor (Ref.)	1.00		1.00	^∗∗^
	Middle	0.76 (0.68–0.84)	< 0.001	0.86 (0.75–0.98)	0.024
	Rich	0.55 (0.49–0.61)	< 0.001	0.70 (0.58–0.85)	< 0.001

Toilet facility	Unimproved (Ref.)	1.00		1.00	
	Improved	1.07 (0.98–1.17)	0.128	0.99 (0.89–1.10)	0.880

Place of residence	Urban (Ref.)	1.00		1.00	
	Rural	1.40 (1.27–1.54)	< 0.001	0.99 (0.82–1.21)	0.939

Sex of household head	Male (Ref.)	1.00		1.00	^∗∗^
	Female	1.17 (1.06–1.30)	0.001	1.21 (1.08–1.35)	0.001

Distance to health facility	Not big problem (Ref.)	1.00		1.00	
	Big problem	1.11 (1.02–1.21)	0.016	0.98 (0.88–1.09)	0.714

Community poverty	Low (Ref.)	1.00		1.00	^∗∗^
	High	1.52 (1.39–1.65)	< 0.001	1.24 (1.05–1.47)	0.014

^∗∗^Significant at multivariable analysis.

The odds of anemia were significantly lower in children aged 24–35 months (AOR = 0.48; 95% CI: 0.34–0.68), 36–47 months (AOR = 0.41; 95% CI: 0.29–0.58), and 48–59 months (AOR = 0.36; 95% CI: 0.25–0.50), indicating that anemia is most severe during childhood. Nutritional deficiencies continued to be powerful predictors: moderate (AOR = 1.22; 95% CI: 1.07–1.40) and severe underweight status (AOR = 1.58; 95% CI: 1.31–1.92) were associated with higher odds of anemia, as was severe stunting (AOR = 1.19; 95% CI: 1.04–1.37). Moreover, children who had experienced fever within the previous 2 weeks had higher odds of anemia (AOR = 1.24; 95% CI: 1.10–1.40). In contrast, female children were less likely to experience anemia than male children (AOR = 0.89; 95% CI: 0.81–0.98).

Children of mothers with at least secondary education were less likely to have anemia (AOR = 0.85; 95% CI: 0.74–0.98), while maternal anemia was linked to a greater likelihood of anemia in children (AOR = 1.39; 95% CI: 1.26–1.53). Household wealth showed a protective gradient, with households in the middle wealth quintile (AOR = 0.86; 95% CI: 0.75–0.98) and rich households (AOR = 0.70; 95% CI: 0.58–0.85) exhibiting lower odds of childhood anemia compared with poor households. Additionally, children from female‐headed households had more likely anemic (AOR = 1.21; 95% CI: 1.08–1.35). Finally, children living in high‐poverty communities had a greater likelihood of anemia (AOR = 1.24; 95% CI: 1.05–1.47).

## 5. Discussion

The study aimed to investigate the spatial distribution and the examining potential determinants of anemia among children aged 6–59 months using data from the 2023/2024 Democratic Republic of Congo Demographic and Health Survey (DRC‐DHS). The analysis comprised 9449 children aged 6–59 months; 6092 (64.5%) had anemia, whereas 3357 (35.5%) did not. This indicates that more than half of under‐five children in the DRC were affected by anemia. This finding was higher than that of studies [34, 51]. The observed clustering and spatial heterogeneity of childhood anemia, with hotspots in the western and northern provinces and cold spots in the east, highlight substantial geographic inequalities that likely reflect underlying differences in socioeconomic conditions, nutrition, and access to health services across the DRC.

A number of socioeconomic, demographic, and health‐related factors, including child age, sex, stunting status, underweight, fever, maternal education, maternal anemia, wealth index, sex of household head, and community poverty, were found to be significantly associated with anemia.

The study revealed that childhood anemia was strongly influenced by the age of the child based on the AORs. Compared with children aged 6–11 months, those aged 24–35, 36–47, and 48–59 months had 52%, 59%, and 64% lower odds of anemia, respectively. This finding is consistent with several studies [52–55]. This is due to older children consuming more iron‐rich complementary foods, improved nutrient absorption, and greater exposure to routine health interventions such as supplementation and deworming [56, 57].

We also found that the sex of the child was a significant determinant of childhood anemia. Female children were less likely to be anemic compared to male children. This finding is consistent with a previous study in Kenya [58] and inconsistent with a previous study in Bangladesh [59]. This is because male children have higher iron requirements for rapid growth and may be more susceptible to infections that contribute to anemia [60].

In addition, we found that stunting status was a significant determinant of childhood anemia. Severely stunted children had higher odds of anemia compared with nonstunted children. This result aligns with the findings from earlier studies [61, 62]. Severe stunting may impair iron absorption and hematologic function, increasing anemia susceptibility [63].

Additionally, another important aspect affecting childhood anemia is underweight condition. Anemia was more common in children who were somewhat underweight than in those who were of normal weight. Anemia was even more common in children who were severely underweight. This finding is consistent with previous studies conducted in Ethiopia [64, 65]. Fever is also an important determinant of childhood anemia. In contrast to children without fever, those who had fever within the 2 weeks preceding the survey were 1.24 times more likely to develop anemia. This result aligns with findings from earlier studies conducted in Nigeria [66] and Southern Ethiopia [67]. This is because infections causing fever can lead to red blood cell destruction, impaired Hb synthesis, and reduced nutrient absorption, increasing the risk of anemia in children [68].

On the other hand, maternal education was discovered to be a crucial factor of childhood anemia. Children whose mothers had secondary education or higher were 15% less likely to be anemic compared with those whose mothers had no formal education, which is consistent with the studies conducted in Tanzania [69] and the studies conducted in SSA [11]. This is because educated mothers are more likely to have better knowledge of child nutrition, feeding practices, hygiene, and healthcare utilization, which helps prevent anemia in their children [70].

This study further demonstrated that maternal anemia is an important factor contributing to childhood anemia. Anemia was more common in children of anemic mothers than in children of nonanemic mothers. The result is in line with the findings from Bangladesh [35], Ethiopia [71], and South Africa [72]. This is because anemic mothers are more likely to have lower iron stores during pregnancy and lactation, which reduces the iron transferred to the child [73].

In addition, wealth index and sex of household head significantly influence childhood anemia in the DRC. Children from middle‐wealth‐index and rich‐wealth households are less likely to experience anemia, respectively, compared with children from poor households. The finding is supported by studies conducted in Ethiopia [71] and Nigeria [66]. This is likely because wealthier households can provide better nutrition, including iron‐rich foods, and have improved access to healthcare services [74].

Determinants of household community–based health insurance membership and healthcare expenditures in rural Awi Zone, Ethiopia: a bivariate logistic regression approach.

Children from female‐headed households were 21% more likely to be anemic compared with those from male‐headed households. The finding is supported by a study conducted in Ghana [75]. This is likely due to limited household income, reduced decision‐making power, and greater caregiving burdens in female‐headed households, which can restrict access to nutritious foods and healthcare for children.

Finally, community poverty is another crucial factor influencing childhood anemia, Compared to children in low‐poverty communities, children in high‐poverty groups were more likely to suffer from anemia. The result is in line with findings [76]. Children in these environments are more susceptible to anemia because they have less access to wholesome diets, clean water, sanitary facilities, and medical care.

## 6. Strengths and Limitations

The results of this study can be applied at the national level because it used nationally representative 2023/2024 DRC‐DHS data. The combination of spatial analysis and multilevel binary logistic regression enabled the identification of anemia hotspots and provided robust estimates while accounting for the hierarchical structure of the data. However, the cross‐sectional design limits the ability to infer causal relationships. Additionally, some crucial variables could not be included due to the use of secondary DHS data, such as dietary intake and malaria infection. Potential recall bias from maternal responses, residual confounding from unmeasured factors, and displacement of DHS cluster coordinates may have affected the accuracy of some findings, particularly the spatial analysis. Despite these limitations, the large sample size and advanced analytical approach strengthen the reliability and relevance of the study findings.

## 7. Conclusion

The findings indicate that childhood anemia among children aged 6–59 months in the DRC is not randomly distributed but exhibits significant spatial clustering, with high‐risk hotspot areas identified in Equateur and Tshuapa provinces. Anemia continues to pose a substantial public health burden among children aged 6–59 months. The burden of anemia was associated with multiple individual, household, and community‐level factors, including child age, sex, stunting, underweight, recent fever, maternal anemia, maternal education, household wealth status, sex of the household head, and community‐level poverty. These findings provide evidence to support geographically targeted and equity‐focused public health strategies that prioritize high‐risk areas and vulnerable populations while addressing nutritional deficiencies, maternal health, and socioeconomic inequalities in the DRC.

**Nomenclature**
AIC: Akaike information criteria
AOR: Adjusted odds ratio
DRC‐DHS: Democratic Republic of Congo Demography and Health Survey
BIC: Bayesian information criteria
CI: Confidence interval
ICC: Intraclass correlation coefficient
MOR: Median odds ratio
OR: Odds ratio
PCV: Proportional change in variance
SSA: Sub‐Saharan Africa
WHO: World Health Organization

## Author Contributions

Z.M.W. conceptualized and designed the study, conducted the data analysis, and prepared the first draft of the manuscript. N.A.B., K.T.G., W.E.S., K.K.H., and E.A.A. contributed to data management and analysis. B.C.J., D.B.B., and R.R.M. contributed to the interpretation of the findings and critically reviewed and revised the manuscript.

## Funding

We did not receive external funds for this research.

## Disclosure

All authors read and approved the final manuscript.

## Ethics Statement

The Democratic Republic of the Congo’s Institutional Review Board (IRB) and the ICF IRB granted ethical approval for the initial survey. This study used secondary, anonymized data from the Demographic and Health Survey (DHS) of the Democratic Republic of the Congo; therefore, informed consent and additional ethical clearance for this secondary analysis were not applicable. http://www.dhsprogram.com.

## Consent

The authors have nothing to report.

## Conflicts of Interest

The authors declare no conflicts of interest.

## Data Availability

The data that support the findings of this study are available upon request from the corresponding author. The data are not publicly available due to privacy or ethical restrictions.
